# A Phase I Clinical Trial of Intrahepatic Artery Delivery of TG6002 in Combination with Oral 5-Fluorocytosine in Patients with Liver-Dominant Metastatic Colorectal Cancer

**DOI:** 10.1158/1078-0432.CCR-24-2498

**Published:** 2025-01-09

**Authors:** Emma J. West, Alain Sadoun, Kaidre Bendjama, Philippe Erbs, Cristina Smolenschi, Philippe A. Cassier, Thierry de Baere, Sophie Sainte-Croix, Maud Brandely, Alan A. Melcher, Fay Ismail, Karen J. Scott, Angela Bennett, Emma Banks, Ewa Gasior, Sarah Kent, Marta Kurzawa, Christopher Hammond, Jai V. Patel, Fiona J. Collinson, Chris Twelves, D. Alan Anthoney, Dan Swinson, Adel Samson

**Affiliations:** 1 Leeds Institute of Medical Research at St. James’s, University of Leeds, Leeds, United Kingdom.; 2 Transgene, Illkirch-Graffenstaden, France.; 3 Institut Gustave Roussy, Villejuif, France.; 4 Medical Oncology, Centre Léon Bérard, Lyon, France.; 5 The Institute of Cancer Research, London, United Kingdom.; 6 Leeds Teaching Hospitals NHS Trust, Leeds, United Kingdom.

## Abstract

**Purpose:**

Effective treatment for patients with metastatic cancer is limited, particularly for those with colorectal cancer with metastatic liver lesions, in which accessibility to numerous tumors is essential for favorable clinical outcomes. Oncolytic viruses (OV) selectively replicate in cancer cells; however, direct targeting of inaccessible lesions is limited when using conventional intravenous or intratumoral administration routes.

**Patients and Methods:**

We conducted a multicenter, dose-escalation, phase I study of vaccinia virus, TG6002, via intrahepatic artery (IHA) delivery in combination with the oral prodrug 5-fluorocytosine to 15 patients with metastatic colorectal cancer.

**Results:**

Successful IHA delivery of replication-competent TG6002 was achieved, as demonstrated by the virus within tumor biopsies. Functional transcription of the *FCU1* transgene indicates viral replication within the tumor, with higher plasma 5-fluorouracil associated with patients receiving the highest dose of TG6002. IHA delivery of TG6002 correlated with a robust systemic peripheral immune response to the virus with activation of peripheral blood mononuclear cells, associated with a proinflammatory cytokine response and release of calreticulin, potentially indicating immunogenic cell death. Gene Ontology analyses of differentially expressed genes reveal a significant immune response at the transcriptional level in response to treatment. Moreover, an increase in the number and frequency of T-cell receptor clones against both cancer antigens and neoantigens, with elevated functional activity, may be associated with improved anticancer activity. Despite these findings, no clinical efficacy was observed.

**Conclusions:**

In summary, these data demonstrate the delivery of OV to tumor via IHA administration, associated with viral replication and significant peripheral immune activation. Collectively, the data support the need for future studies using IHA administration of OVs.

**Translational Relevance**

Very few studies have tested the intrahepatic artery administration of oncolytic viruses. In this study, we delivered TG6002 oncolytic vaccinia virus via the hepatic artery to patients with colorectal cancer liver metastases. We showed successful delivery of TG6002 to the tumor via this approach with the functional activity of the virus-encoded *FCU1* transgene, converting orally administered 5-fluorocytosine to clinically relevant concentrations of the active chemotherapeutic 5-fluorouracil. Innate and adaptive anticancer immune responses were elicited. This study paves the way for future locoregional approaches to the treatment of liver-dominant cancers and to the virus-encoded conversion of prodrugs into active chemotherapeutics as a way to limit systemic chemotherapy toxicity.

## Introduction

Colorectal cancer (CRC) is a leading cause of cancer-associated deaths in Western populations and the third most frequent cause of cancer-related deaths worldwide (1). The 5-year survival rate for localized disease is approximately 91%; however, around 25% to 30% of patients with colorectal cancer develop liver metastases (2), which is associated with a 5-year survival rate of only 13% (3). For these patients, systemic anticancer therapy is the mainstay of treatment, with 5-fluorouracil (5-FU) being commonly employed either as monotherapy or in combination with other cytotoxics. However, 5-FU has limitations including intravenous (i.v.) administration, short half-life, significant systemic toxicity, and drug resistance (4). For patients with liver-dominant metastatic colorectal cancer (mCRC), locoregional therapies offer the prospect of effective treatment while limiting systemic toxicity.

Oncolytic viruses (OVs) are principally immunotherapeutic agents that preferentially replicate in malignant cells, ultimately inducing immunogenic cell death (ICD). OVs can be engineered to express transgenes with immune-stimulating functions or highly specific downstream targets (5). Many engineered OVs have been evaluated in randomized trials, with three currently licensed as standard care (6). One virus that has been tested extensively in the clinical setting is *pexastimogene devacirepvec* (*Pexa-Vec*; JX-594, TG6006), an engineered Wyeth-strain vaccinia virus (7) developed by Transgene and Sillagen. Clinical efficacy as a single agent by intratumoral (i.t.) injection was observed in a dose comparison, randomized study in patients with hepatocellular carcinoma (HCC), in which overall survival was significantly longer for patients in the high-dose group (8). Furthermore, i.v. delivery to the tumor is also possible at a dose of 1 × 10^9^ plaque-forming units (pfu; ref. 9).

TG6002 was developed by engineering the highly oncolytic Copenhagen vaccinia strain (10, 11), incorporating gene modifications to enhance its antitumor activity and clinical impact. Thymidine kinase and ribonucleotide reductase genes are deleted in TG6002, enhancing selective replication in cancer cells (12). In addition, the insertion of the chimeric yeast *FCU1* gene enables the selective conversion of the prodrug 5-fluorocytosine (5-FC) into the cytotoxic 5-FU and 5-fluorouridine monophosphate (13), bypassing the natural resistance of tumor cells to 5-FU alone and reducing systemic toxicity (12). Moreover, TG6002 induces an antitumor immune response involving CD8 T cells and tumor-infiltrating lymphocytes and myeloid cells (14).

TG6002 with 5-FC is a promising combination therapy for cancers that are sensitive to 5-FU. An open-label phase I dose-escalation trial of i.v. TG6002 plus 5-FC was initiated in 2018 (TG6002.02; NCT03724071) in patients with advanced gastrointestinal malignancies. Overall, the combination was well tolerated, and no maximum-tolerated dose (MTD) was observed. Preliminary results indicate effective biodistribution of TG6002 in tumor cells, associated with localized *FCU1* activity (15).

Critical to successful OV therapy is the delivery of the virus to the tumor site. Various routes of administration have been investigated, predominantly i.t. and i.v. Intravenous administration is simple and relatively noninvasive and can achieve systemic delivery of the virus to all vascularized tumors although very high doses are required to achieve sufficient concentration at the tumor site, as the majority is redistributed throughout normal body organs. Intratumoral administration delivers the virus directly to the tumor; however, i.t. injection is limited to radiologically detectable, anatomically and technically injectable lesions although abscopal effects have been reported at distant sites. For patients with liver-dominant cancers, an alternative route of delivery is via selective catheterization of the hepatic artery; indeed, locoregional delivery of chemotherapy via the hepatic artery has been extensively studied (16, 17) and was initially considered for administration of OVs in the early 2000s (18). Intrahepatic artery (IHA) infusion of OVs has the potential to enhance delivery and distribution to multiple tumors across the liver while limiting systemic chemotherapy toxicity. We describe the clinical and translational data from a dose-escalation study of TG6002 via IHA administration plus oral 5-FC. The results show that IHA administration of an OV is clinically achievable and results in the delivery of replication-competent virus to the tumor, expression and clinically relevant activity of the *FCU1* transgene, peripheral activation of the immune system, and potential ICD.

## Patients and Methods

### Study design

**Figure 1.**
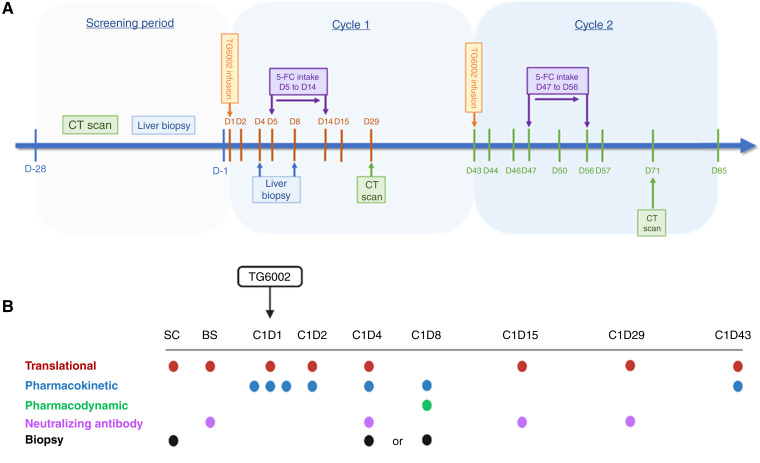
Trial schema and sampling schedule. **A,** Trial schema depicting treatment schedule of two planned cycles of IHA TG6002 and oral 5-FC. **B,** Patient blood and tissue samples taken at various time points prior to and during treatment. Blood samples were specific for downstream analyses: translational (red), pharmacokinetic (blue), pharmacodynamic (green), neutralizing antibody (purple), and tissue biopsies (black). SC, screening; BS, baseline; C, cycle; D, day. (Created with BioRender.com.)

TG6002.03 was an open-label, dose-escalation, 3 + 3 design, phase I study (Eudra-CT 2018-004103-39) conducted in three sites across the UK and France in patients with unresectable colorectal cancer with liver metastases having progressed on or after standard chemotherapy, including at least a fluoropyrimidine, oxaliplatin, and irinotecan or, in the UK only, entering a period of clinical observation following discontinuation of chemotherapy. Patients received up to two cycles of TG6002 combined with oral 5-FC (Fig. 1A). TG6002 was administered via the main hepatic artery, through a catheter inserted into the femoral artery under angiographic assessment, for more than 30 minutes at doses of 1 × 10^6^, 1 × 10^7^, 1 × 10^8^, and 1 × 10^9^ pfu. 5-FC was taken orally from days 5 to 14 at a dose of 50 mg/kg four times daily. A second treatment cycle was to be administered from day 43 in the absence of disease progression or unacceptable toxicity. Dose escalation proceeded after a review of safety data from each cohort by an independent safety review committee. The clinical trial protocol was approved by institutional ethics committees and conducted in accordance with the Declaration of Helsinki. All patients signed an informed consent document prior to study participation.

### Patient samples

Blood and tissue samples were collected and processed using the Translational Cancer Immunotherapy Team quality-assured lab manual, which included standard operating procedures to regulate all processes. Peripheral blood was collected into tripotassium ethylene-diaminetetraacetic acid (K3EDTA) or serum clot-activator vacutainer tubes (both Scientific Laboratory Supplies) and processed within 2 hours of venepuncture, or as soon as possible thereafter. Tumor biopsies collected on day 4 or 8 were placed in formalin or RNAlater (both ThermoFisher) for IHC or PCR analyses, respectively. All sample collection time points are shown in Fig. 1B.

### Isolation of PBMCs, plasma, and serum from whole blood

Serum clot-activator tubes were left for a minimum of 30 minutes after venepuncture. All blood collection tubes were centrifuged for 10 minutes at 2,000 g. Plasma and serum aliquots from the upper layers were stored at −80°C. Peripheral blood mononuclear cells (PBMCs) were isolated by density-gradient centrifugation over Lymphoprep (STEMCELL Technologies) as per the manufacturer’s instructions. Cells were frozen at 1 × 10^7^/mL in 40% (v/v) Roswell Park Memorial Institute medium containing 5 mmol/L L-glutamine and 1 mmol/L sodium pyruvate (all Sigma), plus 50% (v/v) pooled human serum (SeraLab) and 10% (v/v) dimethyl sulfoxide (Sigma). PBMCs were stored in liquid nitrogen.

### Detection of virus in tumor biopsies and plasma

#### qPCR

DNA was extracted from tumor biopsies using DNeasy Blood and Tissue Kits (QIAGEN). Primers (forward 5′-CGA​TGA​TGG​AGT​AAT​AAG​TGG​TAG​GA-3′ and reverse 5′-CAC​CGA​CCG​ATG​ATA​AGA​TTT​G-3′; Integrated DNA Technologies) were used to detect the presence of TG6002.

#### qRT-PCR

qRT-PCR was performed on tumor biopsies and plasma for the detection of viral early *D7R*, viral late *A10L*, and *FCU1* transcripts. RNA was extracted using RNeasy Plus Mini Kits (QIAGEN). The remaining viral and cellular DNA in samples was digested with TURBO DNase (ThermoFisher). Primers and probes for D7R (forward TTT​AGC​GAT​TCA​AAG​TAC​TGC​TTT​TT, reverse GCA​GTG​ACT​TCG​CTG​CCA​TT, and probe FAM-CGAAATGGTAATGCGTATGA), A10L (forward CTT​CAT​ACT​CGC​GAT​CCT​CAA​A, reverse TCG​CCA​ACA​GGT​TAA​AGA​AAT​TAA, and probe ABY-TGGCGCTTCCAAACGTGCAATTT), and *FCU1* (forward TCG​TGG​TCA​CAA​CAT​GAG​ATT​TC, reverse TCT​AAT​CTC​CCA​CAG​TTT​TCC​AAA​G, and probe ABY-TCCGCCACACTACATGGTGAGATCTCC) were used. Detection of the TG6002 viral genome in plasma was performed by Charles River Laboratories, Evreux, using in-house methods. All PCR data were acquired on Applied Biosystems QuantStudio™ 5 Real-Time PCR Systems (ThermoFisher; RRID:SCR_020240) and analyzed using QuantStudio 3D AnalysisSuite Cloud (ThermoFisher; RRID:SCR_020238). The presence of the virus in tumor biopsies was considered positive by RT-qPCR if at least one of the viral mRNAs (*D7R*, *A10L*, or *FCU1*) was detected. RNase-free water was used as a negative control.

Plaque assays were performed by Transgene, France, using in-house methods. Briefly, tissue biopsies were sonicated for 15 seconds at room temperature before incubation with a permissive cell line, Vero (CCL-81; ATCC; RRID:CVCL_0059). Veros (passage number 128 at thawing) were cultured in Dulbecco's modified Eagle's medium (DMEM; Sigma) supplemented with 10% (v/v) fetal calf serum (FCS; ThermoFisher) and 40 mg/L gentamicin (Sigma) for two passages prior to use in plaques assays. Confluent monolayers were incubated with tumor samples for 30 minutes prior to incubation at 37°C under 1% (w/v) agarose for 3 days. Positive infection was determined by the presence of viral plaques. Veros were authenticated in 2014 (by qPCR and epifluorescence microscopy) by Clean Cells and tested negative for *Mycoplasma* infection.

IHC for viral protein was performed by Cerba Research. A polyclonal anti-vaccinia virus antibody (Meridian Life Science; RRID:AB_153134) was used to detect virions; negative control was secondary antibody alone. 3, 3-diaminobenzidine-horseradish peroxidase (DAB-HRP) was used to visualize virus-positive cells. Data from all methods are expressed as positive or negative/below the level of detection. Biopsies from two patients (13 and 15) were not available.

### 5-FU concentrations in serum and tumor tissue

Quantification of 5-FC, 5-FU, and 5-fluoro-β-alanine (F-BAL) levels was performed using liquid chromatography coupled with high-resolution mass spectrometry (Hospices Civils de Lyon) as described previously (19). Serum was evaluated on day 8 after TG6002. Additionally, 5-FU concentrations were measured in available tumor biopsies on day 8 post-TG6002. F-BAL was not measured in cohort 1 serum samples or tumor biopsies due to sample insufficiency.

### Calreticulin ELISA

Patient plasma was analyzed for calreticulin (CRT) by ELISA (ThermoFisher) as per the manufacturer’s instructions. Data are expressed as mean plasma concentration (ng/mL) ± SEM, calculated using a standard curve. Statistical significance was determined using paired two-tailed *t* tests (GraphPad Prism; RRID:SCR_002798) between sample time points (* *P* < 0.05; *n* = 14 patients), dependent on sample availability.

### mRNA expression analysis of patient PBMCs

mRNA sequencing was performed by Novogene as per validated methods. After PCR, the gene expression level was calculated by the number of mapped reads. Statistically significant differentially expressed genes (ssDEG) were defined as ± >2 log_2_ fold change of post-treatment samples compared with baseline with an associated *P*adj < 0.05. Data from six patients, across three cohorts, are shown for ssDEGs for C1D2 and C1D15. *P*adj values were transformed into −log_10_(*P*adj) values, which were plotted against log_2_ fold change values in volcano plots. Volcano plots depict all DEGs, not just ssDEGs, which are upregulated, downregulated, or unchanged for three patients at C1D2 and C1D15 compared with BS. ssDEG lists were analyzed using the Search Tool for Retrieval of Interacting Genes/Proteins (STRING) database (http://string-db.org; RRID:SCR_005223) to identify potential interactions between the genes and their reported biological function(s). Interaction confidence scores (ICS) were assigned to each protein association and ranked from 0 to 1, in which 1 is most likely to be accurate and 0 is least likely to be correct. An ICS of 0.5 indicates that every second interaction may be a false positive; therefore, only ssDEGs with an ICS of >0.5 were used for visualization and analysis. Gene Ontology (GO) biological functions were assessed at the ssDEG level, in which common genes were detected across multiple patients. These nine commonly expressed ssDEGs were analyzed independently in the STRING database to identify highly responsive signaling pathways following treatment.

### Immunophenotyping

PBMCs were analyzed for specific activation/immune checkpoint molecules. Briefly, PBMCs were stained for CD3 (HIT3a/FITC; RRID:AB_395745), CD4 (RPA-T4/APC-H7; RRID:AB_1645478), CD8 (RPA-T8/Alexa 700; RRID:AB_396953), CD56 (B159/PE-Cy7; RRID:AB_396853), CD19 (SJ25C1/APC-H7; RRID:AB_1645470), CD14 (M5E2/FITC; RRID:AB_395798), CD69 (FN50/APC; RRID:AB_398602), programmed cell death-ligand 1 (PD-L1; MIH1/PE-CF594; RRID:AB_2738400), programmed cell death protein 1 (PD-1; MIH4/PE; RRID:AB_647199), T-cell immunoglobulin and mucin-domain containing-3 (TIM-3; 7D3/BV786; RRID:AB_2741100), OX40 (L106/BV421; RRID:AB_2742558), CD40 (5C3/BUV395; RRID:AB_2739110), and CD25 (M-A251/BB700; RRID:AB_2744335; all BD Biosciences) plus CD40 ligand (L) (24-31/BV786; RRID:AB_2572187) and cytotoxic T-lymphocyte-associated protein 4 (CTLA-4; BNI3/BV605; RRID:AB_2632779; both BioLegend). Fluorescence minus one (FMO) was used as a negative control. Data were acquired on a CytoFLEX LX and analyzed using CytExpert software (RRID:SCR_017217) (both Beckman Coulter). Positive expression of markers was used to calculate fold-change differences ± SEM in expression from baseline samples. Paired two-tailed *t* tests (GraphPad Prism) were used to determine statistical significance between samples (* *P* < 0.05; *n* = 9 patients), dependent on sample availability.

### IHC for PD-L1

Formalin-fixed, paraffin-embedded tissue biopsies were stained with rabbit anti-human PD-L1 antibody (1:500; Abcam; RRID:AB_2884993) and ImmPRESS HRP anti-rabbit IgG (peroxidase) secondary antibody (Vector Laboratories; RRID:AB_2336529). Positive staining was visualized using ImmPACT DAB Peroxidase (HRP) Substrate kit (Vector Laboratories). Control sections contained no primary antibody. Digital images were acquired at 20× magnification and quantified using QuPath software (RRID:SCR_018257). Data are expressed as cells positive for PD-L1 per mm^2^ for *n* = 7 patients.

### Enzyme-linked immunosorbent spot (ELISpot)

Briefly, triplicates of 1 × 10^5^/well PBMCs were incubated in the presence of either 2 μg/mL of overlapping peptide pools for carcinoembryonic antigen (CEA) or cytomegalovirus/Epstein-Barr virus/influenza (CEF; positive control) (both Cambridge Biosciences). Negative control was media alone; 10 pfu/cell TG6002 was used to assess response to treatment. IFNγ secretion from activated T cells was detected using a matched paired antibody kit (Mabtech). Spot-forming units (SFU) were visualized using 5-bromo-4-chloro-3-indolyl phosphate/nitroblue tetrazolium substrate (MabTech). Images were captured and quantified using an S6 FluoroSpot analyzer (Cellular Technology Limited). Data are presented as mean fold-change SFU per well ± SEM for post-treatment samples compared with baseline, for *n* = 6 patients, dependent on sample availability.

### TCR-β sequencing

T-cell receptor (TCR) β sequencing was performed by Adaptive Technologies using a “survey” resolution to generate data from productive rearrangements only, which were exported from the immunoSEQ Analyzer (Adaptive Technologies) for further analysis. Complementary-determining region 3 (CDR3) sequences were input into the McPAS-TCR database (RRID:SCR_026024) and matched to known human TCR sequences. TCRs in each patient sample that matched known cancer antigen or neoantigen epitopes were identified; these were counted and their total productive frequency was calculated. Data are expressed as the number of TCRs matching cancer antigens/neoantigens (*x*-axis) against the productive frequency of TCRs matching cancer antigens/neoantigens (*y*-axis) for all available PBMC samples.

### Data availability

The mRNA sequencing FASTQ files are available in the Sequence Read Archive database (accession number PRJNA1192197). TCR sequencing data can be accessed via the immuneACCESS server using the following link: https://doi.org/10.21417/EW2024S. All data generated in this study are available upon request from the corresponding author.

## Results

### Patient characteristics

In total, 20 patients were screened; of these, 15 patients were entered into the study across three sites (Supplementary Table S1) and received at least one infusion of TG6002. The mean age was 61 years (range, 37–78 years), comprising 11 males and four females, which is a slightly younger age range and a higher male:female ratio compared with global patient demographics (Supplementary Table S2). Patients had mismatch repair (MMR) proficient cancers. Included patients had either progressed or were intolerant to both oxaliplatin- and irinotecan-based chemotherapy or were undergoing a period of observation following a course of chemotherapy. The primary tumor was in the colon in 11 patients and the rectum in four patients. The mean time from initial diagnosis to trial entry was 36.5 months (range, 8.1–89.8 months), and the patients had received a mean of 3.3 prior lines of antineoplastic therapy (range, 1–7), including adjuvant lines. Thirteen patients completed the trial; two patients withdrew prematurely, one with dose-limiting toxicity (DLT) and one because of palliative best supportive care. No patient deaths were related to TG6002 and/or 5-FC; of the 13 patients completing the trial, the cause of death for 12 patients was a progression of the underlying disease and one case of non-study treatment-related pneumonia occurred 22.3 months after inclusion.

### Patient exposure

Of the 15 patients entered, three were treated in each of the 3 + 3 design cohorts (1, 2, and 3), with six treated in cohort 4 (Supplementary Table S3), as planned. Fourteen patients received only a single cycle of treatment due to the progression of their disease, and one received two cycles; each cycle being a single dose of TG6002 via IHA infusion on day 1 followed by 10 days of oral 5-FC on days 5 to 14 (Fig. 1A). All infusions were fully administered. In 13 patients, the whole liver was perfused, whereas two patients had partial liver perfusion because of anatomical considerations. One patient did not receive 5-FC, having withdrawn from the trial on day 1 after the TG6002 infusion and before receiving 5-FC. Thirteen of the remaining 14 patients received their complete 10-day course of 5-FC, but one patient discontinued 5-FC after 9 days.

### Safety data

Overall, 14 patients (93.3%) experienced at least one study treatment–related adverse event (AE), of whom 13 (86.7%) experienced at least one AE related to TG6002 and nine (60.0%) experienced at least one AE related to 5-FC (Tables 1 and 2).

**Table 1.** AEs summarized by relationship to grade.

	Grade 1	Grade 2	Grade 3
	*n* (%)	*n* (%)	*n* (%)
Patient with at least one AE	13 (86.7)	10 (66.7)	5 (33.3)
Blood and lymphatic system disorders: anemia	0 (0.0)	1 (6.7)	1 (6.7)
Cardiac disorder: myocardial infarction	0 (0.0)	0 (0.0)	1 (6.7)
Gastrointestinal disorders:	8 (53.3)	5 (33.3)	1 (6.7)
Nausea	1 (6.7)	2 (13.3)	0 (0.0)
Abdominal pain	1 (6.7)	2 (13.3)	0 (0.0)
Diarrhea	4 (26.7)	0 (0.0)	1 (6.7)
Vomiting	2 (13.3)	0 (0.0)	1 (6.7)
General disorders:	7 (46.7)	9 (60.0)	1 (6.7)
Pyrexia	4 (26.7)	4 (26.7)	1 (6.7)
Fatigue	2 (13.3)	6 (40.0)	0 (0.0)
Chills	2 (13.3)	1 (6.7)	0 (0.0)
Influenza-like illness	0 (0.0)	2 (13.3)	0 (0.0)
Hepatobiliary disorders: hepatic pain	1 (6.7)	1 (6.7)	0 (0.0)
Infections and infestations: cystitis	1 (6.7)	0 (0.0)	0 (0.0)
Investigations:	8 (53.3)	2 (13.3)	1 (6.7)
Aspartate aminotransferase increased	2 (13.3)	1 (6.7)	0 (0.0)
Metabolism and nutrition disorders:	4 (26.7)	0 (0.0)	0 (0.0)
Decreased appetite	2 (13.3)	0 (0.0)	0 (0.0)
Hyperkalemia	1 (6.7)	0 (0.0)	0 (0.0)
Hypokalemia	1 (6.7)	0 (0.0)	0 (0.0)
Hyponatremia	1 (6.7)	0 (0.0)	0 (0.0)
Musculoskeletal and connective tissue disorders:	2 (13.3)	2 (13.3)	0 (0.0)
Myalgia	1 (6.7)	1 (6.7)	0 (0.0)
Arthralgia	1 (6.7)	0 (0.0)	0 (0.0)
Back pain	0 (0.0)	1 (6.7)	0 (0.0)
Pain in extremity	0 (0.0)	1 (6.7)	0 (0.0)
Nervous system disorders:	4 (26.7)	1 (6.7)	0 (0.0)
Headache	4 (26.7)	1 (6.7)	0 (0.0)
Psychiatric disorders:	1 (6.7)	2 (13.3)	0 (0.0)
Anxiety	1 (6.7)	1 (6.7)	0 (0.0)
Depression	0 (0.0)	1 (6.7)	0 (0.0)
Respiratory, thoracic, and mediastinal disorders:	1 (6.7)	1 (6.7)	0 (0.0)
Dyspnea	1 (6.7)	1 (6.7)	0 (0.0)
Skin and subcutaneous tissue disorders:	2 (13.3)	1 (6.7)	0 (0.0)
Hyperhidrosis	1 (6.7)	1 (6.7)	0 (0.0)
Night sweats	1 (6.7)	0 (0.0)	0 (0.0)
Vascular disorders: hypertension	0 (0.0)	1 (6.7)	2 (13.3)

**Table 2.** AEs summarized by relationship to TG6002 and 5-FC.

	1 × 10^6^ pfu	1 × 10^7^ pfu	1 × 10^8^ pfu	1 × 10^9^ pfu	Overall
	*n* (%)	*n* (%)	*n* (%)	*n* (%)	*n* (%)
AE (all grades)	3 (100.0)	3 (100.0)	2 (66.7)	6 (100.0)	14 (93.3)
AE (grade 3)	1 (33.3)	0 (0.0)	0 (0.0)	4 (66.6)	5 (33.3)
AE related to study treatment	3 (100.0)	3 (100.0)	2 (66.7)	6 (100.0)	14 (93.3)
AE related to TG6002	3 (100.0)	2 (66.7)	2 (66.7)	6 (100.0)	13 (86.7)
AE related to 5-FC	2 (66.7)	3 (100.0)	1 (33.3)	3 (100.0)	9 (60.0)
TG6002-related AEs:					
Pyrexia	1 (33.3)	0 (0.0)	1 (33.3)	6 (100.0)	8 (53.3)
Fatigue	1 (33.3)	2 (66.7)	0 (0.0)	1 (16.7)	4 (26.7)
Chills	0 (0.0)	0 (0.0)	0 (0.0)	3 (50.0)	3 (20.0)
Upper abdominal pain	1 (33.3)	1 (33.3)	0 (0.0)	1 (16.7)	3 (20.0)
Nausea	1 (33.3 )	0 (0.0)	0 (0.0)	1 (16.7)	2 (13.3)
Influenza-like illness	1 (33.3)	0 (0.0)	0 (0.0)	1 (16.7)	2 (13.3)
Aspartate aminotransferase increased	0 (0.0)	0 (0.0)	0 (0.0)	1 (16.7)	1 (6.7)
Myocardial infarction	0 (0.0)	0 (0.0)	0 (0.0)	1 (16.7)	1 (6.7)
5-FC-related AEs:					
Diarrhea	0 (0.0)	2 (66.7)	0 (0.0)	1 (16.7)	3 (20.0)
Fatigue	1 (33.3)	2 (66.7)	0 (0.0)	0 (0.0)	3 (20.0)
Nausea	1 (33.3)	0 (0.0)	0 (0.0)	1 (16.7)	2 (13.3)
Pyrexia	1 (33.3)	0 (0.0)	0 (0.0)	1 (16.7)	2 (13.3)
Vomiting	0 (0.0)	1 (33.3)	0 (0.0)	1 (16.7)	2 (13.3)
Chills	0 (0.0)	0 (0.0)	0 (0.0)	1 (16.7)	1 (6.7)
Upper abdominal pain	0 (0.0)	1 (33.3)	0 (0.0)	0 (0.0)	1 (6.7)

Eight grade 3 AEs were observed in five patients, including myocardial infarction (MI), diarrhea, vomiting, pyrexia, and increased aspartate aminotransferase related to study treatment (Tables 1 and 2), plus hypertension and anemia unrelated to treatment. Overall, no AEs greater than grade 3 were reported. Grade 3 TG6002-related AEs included pyrexia and MI in one cohort 4 patient, which constituted a DLT (Tables 1 and 2). This patient acquired an asymptomatic COVID-19 infection near the time of TG6002 infusion, precipitating a supraventricular tachycardia. Four hours following the TG6002 infusion, the patient had a fever and tachycardia that peaked at 40.1°C and 148 bpm. On day 2, an electrocardiogram showed negative T waves, and the troponin I level was increased at 1,555 pg/L (normal level < 45). A coronary angiography was performed on day 3 showing a monotruncular lesion of the anterior interventricular artery leading to coronary stent insertion and successful revascularization. No cases of vesicular or pustular skin or mucosal lesions were reported following TG6002 infusion. Grade 3 AEs related to 5-FC included diarrhea and vomiting in one cohort 4 patient. Aspartate aminotransferase increase occurred in three patients: grade 1 in two patients, with one case related to TG6002, and grade 2 in one patient. Hypertension was reported for three patients: grade 2 in a cohort 2 patient and grade 3 in a patient each in cohorts 1 and 4. Despite being assessed as not related to study treatment, this resulted from antihypertensives being withdrawn during TG6002 administration.

### Trial endpoints and objective efficacy data

No patients had an objective response based on a 10-week disease control rate according to RECIST version 1.1. The primary objective of the maximum feasible dose was 1 × 10^9^; MTD was not reached. Secondary objectives of safety and tolerability were achieved; in addition, viral shedding was not evident in saliva, urine, or feces. Median progression-free survival was 1.05 months, with a range of 0.0 to 2.3 (in which 0.0 relates to the MI reported previously), which is very short because of the timing of the CT scan 4 weeks after TG6002 infusion ahead of the planned second TG6002 infusion. Median overall survival was 5.4 months (range, 1.6–22.3). Although a minority of patients had progressive disease localized within the liver, the majority had indications of both intra- and extrahepatic progressive disease; elevations in circulating CEA levels, compared with baseline levels, were also observed at later time points.

### Detection of TG6002 and *FCU1* transgene activity in tumor biopsy

**Figure 2.**
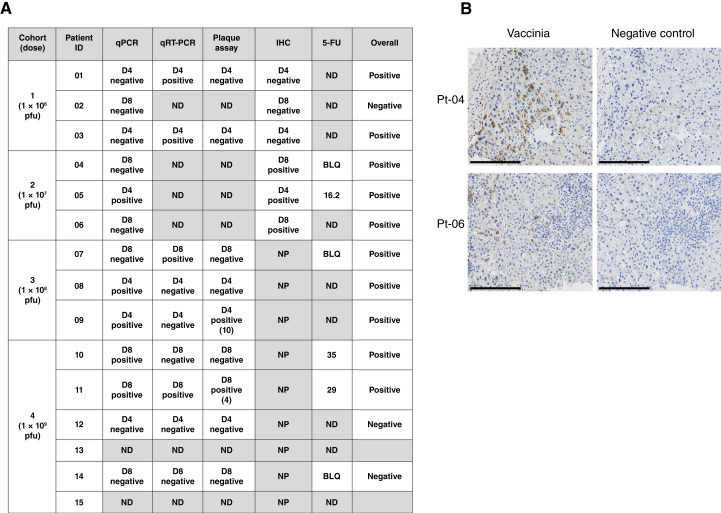
Detection of TG6002 in patient tumor biopsies. **A,** Table summary of data collated from assays to detect the presence of TG6002 in tumor biopsies: qPCR and qRT-PCR to detect viral nucleic acids, plaque assay to detect replication-competent virus (infectious virus particles per biopsy), IHC to determine the presence of virus protein, and 5-FU (pg/mg) to demonstrate transgene activity. Samples are designated as either positive or negative/below the limit of detection for each assay. **B,** Representative examples of positively stained tumor cells by IHC for patients 04 (day 4) and 06 (day 8) (left; brown DAB) and negative control (right; secondary antibody alone). Scale bars, 200 μm. BLQ, below the limit of quantification; D4, day 4; D8, day 8; ND, not done due to insufficient sample; NP, not planned.

Blood and tissue samples for translational analyses were taken as outlined in the trial schedule (Fig. 1B). As only one patient received a second cycle of treatment, the translational assays for all patients were performed on samples obtained during the first cycle only. Tumor biopsies obtained at screening and after treatment (day 4 or 8) were examined for the presence of the TG6002 virus or for the activity of the viral *FCU1* transgene (Fig. 2). Viral DNA by qPCR was detected in five of 13 evaluable tumor samples, predominantly in biopsies from patients who received a higher viral dose than patients in earlier cohorts, both at days 4 and 8 (Fig. 2A). Furthermore, the virus was detected by qRT-PCR in four of nine patients with suitable biospecimens. Plaque assays indicated live replicating TG6002 in two of nine tumors. Viral protein was detected by IHC in three of six evaluable biopsies: one on day 4 and two on day 8, with representative examples shown in Fig. 2B. The active conversion of 5-FC to its metabolite, 5-FU, was detected in three of six evaluable post-treatment biopsies, predominantly in tumors of patients in later cohorts, suggesting that a higher virus dose is required for TG6002 activity within tumor. One patient in cohort 2 (one out of two available biopsies) had 16.2 pg of 5-FU/mg of tumor tissue, whereas two patients in cohort 4 (two out of three available biopsies) had 35 and 29 pg of 5-FU/mg of tumor tissue. Overall, there was evidence of virus infection and viral replication in 10 of 13 patients’ tumors.

### Detection of TG6002 and *FCU1* transgene activity in plasma

**Figure 3.**
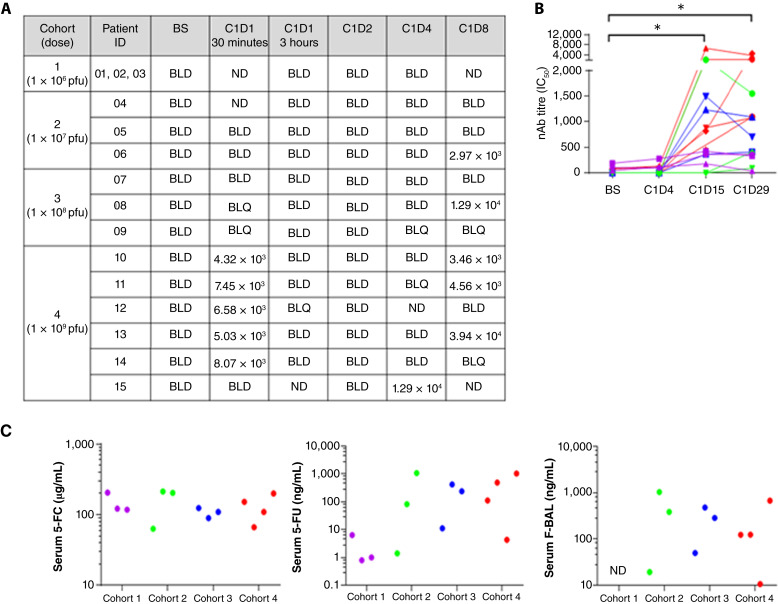
Detection of TG6002, nAb, 5-FC, 5-FU, and F-BAL in plasma following treatment. **A,** Plasma samples analyzed by qPCR to detect TG6002 following treatment. Values stated are the number of copies per milliliter (c/mL). **B,** Serum nAb titers (IC50) for *n* = 15 patients, sample availability–dependent. * *P* < 0.05, paired *t* test. **C,** Serum 5-FC, 5-FU, and F-BAL concentrations at day 8 following treatment (*n* = 13 patients), sample availability–dependent. Cohort 1: purple, cohort 2: green, cohort 3: blue, and cohort 4: red. BLD, below limit of detection; BLQ, below the limit of quantification; C, cycle; D, day; nAb, neutralizing antibody; ND, not done due to insufficient sample.

Despite positive detection in tumors, TG6002 was not found in the vast majority of plasma samples from cohorts 1 to 3, with the exception of one patient in cohort 2 and one in cohort 3, both on day 8, indicating active virus replication (Fig. 3A). In contrast, in the highest dose cohort, TG6002 was detected in plasma 30 minutes post-infusion in five of six patients, followed by undetectable levels indicating rapid clearance. A rebound of circulating TG6002 was observed in one patient on day 4 and three patients on day 8, again indicating active virus replication. Titers of neutralizing antibody (nAb) against TG6002 significantly increased following treatment in all patients (*P* < 0.05), with a trend for higher titers in the highest dose cohort (Fig. 3B).

Serum levels of 5-FC, 5-FU, and the catabolite F-BAL were measured 8 days after exposure to TG6002. Although serum 5-FC concentrations were comparable across all cohorts, patients who received higher virus doses had higher levels of circulating 5-FU than those in cohort 1, indicating replication of TG6002 (Fig. 3C). F-BAL was detected in plasma from all patients from cohorts 2 to 4, where samples were available. Similar to the tumor data, there was evidence of viral presence/replication in serum in all evaluable patients.

### Host response to TG6002/5-FC

**Figure 4.**
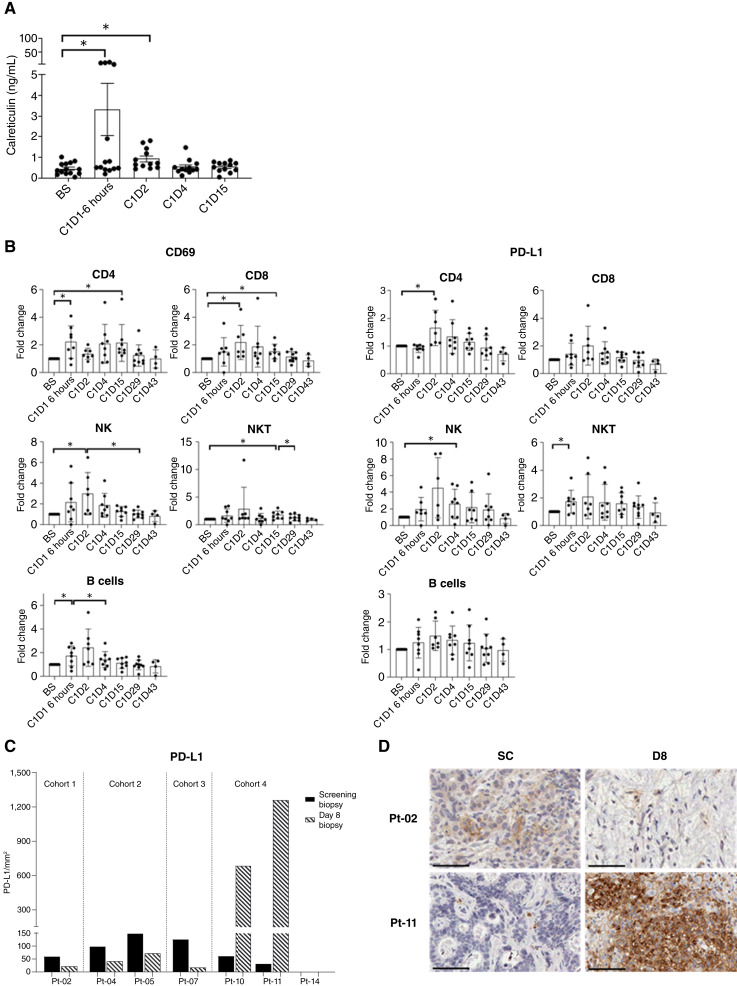
Activation of patient peripheral immune responses following treatment. **A,** CRT concentration (ng/mL) in patient plasma measured by ELISA. Data are expressed as the mean ± SEM; *n* = 14 patients, sample availability–dependent. * *P* < 0.05, paired *t* test. **B,** Immunophenotyping of patient PBMCs for expression of CD69 and PD-L1. Relevant cell populations are depicted for each plot. Data are expressed as the mean fold change ± SEM; *n* = 9 patients. * *P* < 0.05, paired *t* test. **C,** Day 8 tumor PD-L1 expression by IHC expressed as positive cells per square millimeter in screening (black bars) and day 8 post-infusion (hatched bars) biopsies, with representative examples of patients from cohorts 1 and 4 (**D**): screening, left, and day 8, right. Positive staining by DAB (brown); scale bars, 50 μm. SC, screening; BS, baseline; D, day.

The peripheral immune response to IHA infusion of TG6002 was assessed using serial blood samples from patients. CRT was investigated as an indicator of ICD following treatment. CRT concentration in patient plasma significantly increased following TG6002 infusion (Fig. 4A); a peak was detected at 6 hours post-treatment (*P* < 0.05), which remained higher than pre-treatment levels on day 2 (*P* < 0.05), potentially indicating a peak in ICD.

Immunophenotyping of PBMCs revealed CD69 upregulation, an early activation marker, 6 to 24 hours post-infusion on cell populations including CD4^+^ and CD8^+^ T cells, natural killer (NK) cells, natural killer T (NKT) cells, and B cells (Fig. 4B). An increase in PD-L1 expression was also observed, as exemplified on NK cells (Fig. 4B) alongside other immune checkpoint molecules, such as PD-1, TIM-3, and OX40 (Supplementary Fig. S1A). In addition, elevation in CD40L on T cells and NK(T) cells (Supplementary Fig. S1B) and an associated increase in its receptor (CD40) on both monocytes and B cells (Supplementary Fig. S1C) were found, indicating an enhanced capacity for the maturation of antigen-presenting cells. In contrast, a decrease in both CD25 and CTLA-4 on the surface of T cells was apparent, which appeared to be prolonged over time, indicating reduced regulatory T cell functions (Supplementary Fig. S1D).

IHC on tumor biopsies (Fig. 4C) sampled before and after infusion showed low-level PD-L1 expression at baseline and a small reduction in the level of PD-L1 in the tumor by day 8 in patients receiving a lower virus dose (cohorts 1–3). However, with the highest dose (cohort 4), there was a substantial increase in the expression of PD-L1 following virus infusion, reflecting the PBMC data. Representative examples for patients in cohorts 1 and 4 are shown, indicating the extent of cells positive for PD-L1 in patients who received the higher viral load (Fig. 4D).

**Figure 5.**
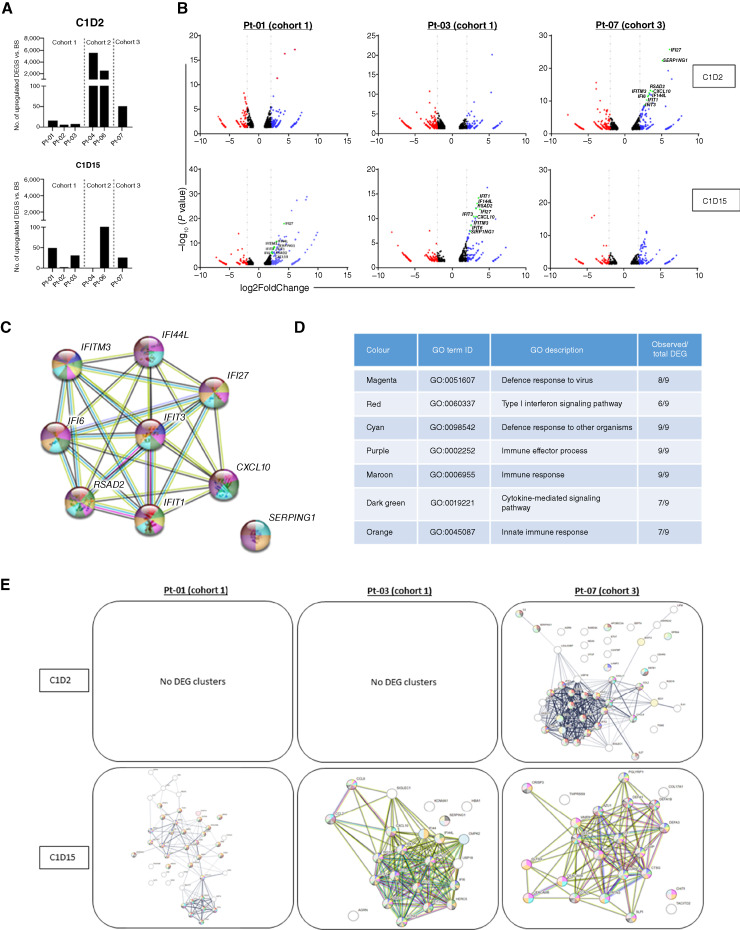
mRNA sequencing of patient PBMCs revealing clustering of DEGs involved in immune activation and response pathways to treatment. **A,** The number of upregulated ssDEGs in the post-treatment samples relative to pretreatment controls for days 2 and 15 post-infusion. **B,** Volcano plots for three patients that highlight nine commonly expressed ssDEGs (red: downregulated DEGs, blue: upregulated DEGs, black: nonsignificant changes to DEGs, and green: specific highlighted ssDEGs). **C,** Cluster plot of the nine commonly expressed ssDEGs in three patients which correspond to (**D**) immune-related GO pathways. **E,** GO analysis showing cluster plots of all upregulated ssDEGs in three patients at days 2 and 15. DEGs are defined as up-/downregulated from baseline, *P*adj < 0.05; ssDEGs are defined as ± >2 log_2_ fold change and *P*adj < 0.05.

mRNA sequencing was used to characterize the effects of TG6002/5-FC treatment at the transcriptional level (Fig. 5). A greater number of ssDEGs were increased at day 2 in PBMCs from patients in cohorts 2 and 3 compared with patients in cohort 1 (Fig. 5A). Patients from cohort 1, who did not show elevated ssDEGs by day 2, had a greater number of ssDEGs on day 15, potentially indicating a delayed response to replicating virus or to 5-FC and its metabolites, including 5-FU. Volcano plots (Fig. 5B) show the pattern of all DEGs in three patients. Nine commonly expressed genes were significantly upregulated in response to TG6002 in three patients, namely, *CXCL10*, *IFIT1*, *IFIT3*, *IFI27*, *IFI44L*, *IFITM3*, *IFI6*, *RSAD2*, and *SERPING1*, all of which are involved in immune-related signaling pathways. Although these specific ssDEGs were evident by day 2 in Pt-07 (cohort 3), an increase in expression was only apparent in patients from cohort 1 (Pt-01 and Pt-03) by day 15. GO analysis of all upregulated ssDEGs generated cluster plots depicting pathways in which the DEGs are highly involved (Fig. 5C–E). Cluster analysis of the nine commonly expressed ssDEGs in the three specified patients (Fig. 5C) revealed a number of pathways highly relevant to immune responses, type I interferon signaling, and, more specifically, response to the virus (Fig. 5D). Each pathway identified had a significant proportion of the nine ssDEGs involved, as indicated. More widely, the predominant clustering of all ssDEGs highlights several immune-related signaling pathways. Pathway clustering was apparent by day 2 in the cohort 3 patient in comparison with day 15 in cohort 1 patients (Fig. 5E). A greater extent of clustering mirrors both the enhanced number of ssDEGs and their earlier appearance following TG6002 infusion, as previously observed (Fig. 5A).

**Figure 6.**
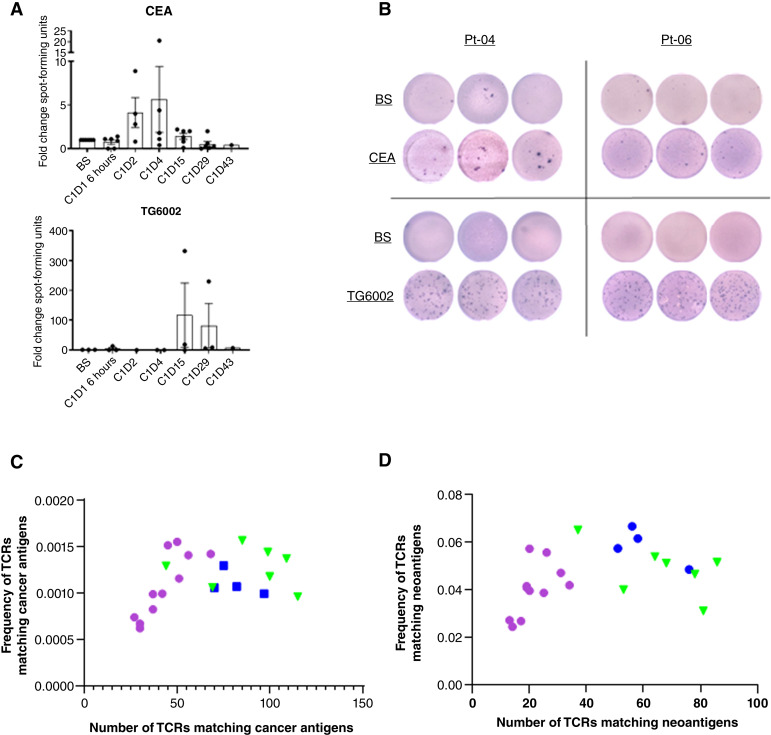
Adaptive T-cell responses to treatment. T-cell function was assessed by ELISpot assay, which showed an IFNγ-release response to CEA and TG6002. **A,** Data expressed as the mean fold change ± SEM SFU of post-treatment samples compared with baseline. *n* = 6 patients, sample availability–dependent. **B,** Representative examples of SFU from two patients showing BS and post-treatment samples, in triplicate, depicting responses to CEA and TG6002. **C** and **D,** TCRβ sequencing examined the T-cell clonal response to TG6002 infusion. CDR3 sequences were matched to known TCRs using the McPAS database. TCRs matched to cancer antigen epitopes (**C**) and neoantigen epitopes (**D**) are shown; data are expressed as number of total matched TCRs (*x*-axis) vs. the sum of productive frequencies of matched TCRs (*y*-axis). Cohort 1; purple, cohort 2: green, and cohort 3: blue. SFU, spot-forming units; CDR3, complementary-determining region 3; BS, baseline.

The adaptive T-cell response to virus infusion was examined by ELISpot assay against a tumor-associated antigen (TAA; CEA) and TG6002 (Figs. 6A and B). CEA-specific T-cell responses were detected by day 4, likely indicating enhanced activation of pre-existing CEA-specific T-cell clones. In contrast, the appearance of TG6002-specific T cells occurred later, at day 15 (Fig. 6A). Furthermore, only patients in the later cohorts (2 and 3) elicited TG6002-specific T-cell responses, presumably due to a higher virus load. Representative wells showing IFNγ responses to CEA and TG6002 are depicted for two patients (Fig. 6B).

TCRβ sequencing of patient tumor and PBMCs at all available time points was also performed. T-cell clonal response to treatment revealed CDR3 sequences matched to cancer antigens (Fig. 6C) and specifically to neoantigens (Fig. 6D), which showed greater frequencies in later cohorts treated with higher viral doses than in earlier lower-dose cohorts.

## Discussion

In total, 15 patients received an IHA infusion of TG6002 plus oral 5-FC as part of a dose-escalation phase I study highlighting the feasibility of this locoregional route of OV delivery in the treatment of liver tumors. IHA delivery of TG6002 was clinically feasible and safe, with the MTD not reached. Dose escalation proceeded as per the protocol, with six patients receiving the highest intended dose of 1 × 10^9^ pfu. Only one patient, in cohort 4, had a DLT that consisted of the aforementioned MI. Disappointingly, no patients experienced clinical or radiological tumor responses, with almost all patients showing continued disease progression one month-post TG6002 delivery. The reasons for this could include the heavily pre-treated population; patients in this trial had all exhausted standard chemotherapy options, meaning that their cancers are likely 5-FU-resistant. Patients in this trial were not selected on the basis of MMR tumor status, and no tumors were known to be MMR-deficient within the recruited cohort. It is likely that all or the majority of recruited patient tumors were MMR-proficient and relatively resistant to immunotherapy so less likely to benefit from OV therapy.

Successful delivery of TG6002 to tumor lesions was achieved via the IHA route. Viral persistence in tumor biopsies sampled post-treatment was evident from a number of analyses, including qPCR, RT-qPCR, plaque assay, IHC, and transgene activity, with the vast majority of patients exhibiting positive detection by one or more methods, despite only limited tissue from a core biopsy being available for analysis. Neutralizing antibodies against TG6002 developed at low levels following infusion, reaching peak titers by day 15 or 29, indicating a humoral immune response to IHA delivery. It is unknown how the presence of low-level nAb might affect the repeated delivery of TG6002 via IHA; further investigation of locoregional oncolytic vaccinia virus therapy for immunotherapy-sensitive tumors is merited.

Functional transcription of the *FCU1* transgene, indicative of a replicating virus, was evident from pharmacokinetic analyses. Serum 5-FU concentrations ranged from 1 to 1,072 ng/mL across all cohorts with significantly elevated levels at higher dose cohorts. Tumor 5-FU titers were detectable in the higher treatment doses, with two patients who received the highest dose of virus having concentrations exceeding 25 pg/mg of tissue. The range of 5-FU concentrations from 16 to 35 pg/mg of tissue compared favorably with that of 5.9 ± 0.9 pg/mg reported in tumor tissue of patients with HCC treated with an oral prodrug of 5-FU (20) and were close to the mean 5-FU concentration of 56.6 pg/mg after i.t. injection of a nonpropagative vaccinia virus expressing *FCU1* in combination with oral 5-FC (21). Of interest, i.v. administered 5-FU can result in higher serum levels of 5-FU, with targeted serum concentrations of 2,500 to 3,000 ng/mL (22, 23), compared with a median of 82 ng/mL detected in our patients receiving oral 5-FC. Therefore, maximizing 5-FU concentrations in the tumor tissue using TG6002/oral 5-FC combination allows direct targeting of malignant cells while minimizing systemic toxicity. Although 5-FU might diffuse from the higher concentrations produced within the tumor microenvironment, the levels should remain higher where a therapeutic effect is desirable. Higher serum concentrations of 5-FU, as experienced during standard i.v. delivery can be problematic, as side effects can be very significant, and many patients are unable to tolerate repeated cycles. Moreover, there is frequently rapid development of resistance to 5-FU alone when administered via an i.v. route.

Despite an apparent lack of clinical efficacy, early peripheral blood immune cell responses indicated immune activity resulting from the combination therapy; in addition to promoting an antitumor response, this may also represent a virally driven immune response to pave the way for both direct tumor lysis and abscopal effects through immune modulation and 5-FU activation. CRT plasma concentrations increased shortly after TG6002 infusion, peaking 6 hours post-treatment and remaining elevated up to 24 hours. CRT is an endoplasmic reticulum–associated chaperone protein ubiquitously expressed intracellularly but also released from cells undergoing ICD (24, 25) and is one of the main hallmarks of ICD in malignant disease. ICD is a unique class of regulated cell death that elicits antigen-specific adaptive immune processes via the release of damage-associated molecular patterns (DAMPs), of which CRT is a key component. The overall role of ICD and DAMP release is the recruitment of antigen-presenting cells to the site of dying tumor cells, in order to promote antigen uptake and processing, prior to cross-presentation to T cells to initiate a tumor-specific immune response.

Associated with the occurrence of ICD, mRNA sequencing revealed a significant response of immune cells at the transcriptional level. A considerable elevation in the number of ssDEGs was apparent in patients who received the highest doses of TG6002. These ssDEGs formed clusters representing immune-related pathways in patients receiving higher virus doses at earlier time points than patients receiving lower doses, suggesting greater immune activation at higher doses of the virus. GO annotations revealed signaling pathways associated with response to virus involving IFN-stimulated genes and, subsequently, immune activation. Specifically, nine ssDEGs were predominantly upregulated in multiple patients and demonstrated to be highly relevant in the aforementioned pathways.

Immunophenotyping of patient PBMCs evidenced immune cell activation across multiple cell populations. CD69, an early activation marker, was elevated shortly after TG6002 infusion, as was the immune checkpoint ligand PD-L1, a common marker for immune activation and exhaustion following OV therapy (26). PD-L1 expression following virus therapy is associated with an anti-tumor immune response driven by IFNs and other inflammatory molecules (27). Furthermore, increased PD-L1 expression by both peripheral and tumor-infiltrating T cells has been associated with a better prognosis for immune checkpoint blockade (28, 29). Likewise, PD-1 and TIM-3 expression are indicative of inflammatory cytokine signaling, including IL-12, IL-15, IL-18, and IFNγ (30, 31). Elevated expression of OX40 on NK cells is broadly associated with activation, in line with previous data (32). Engagement of the CD40/CD40L complex is a secondary activation signal required for T-cell activation and is associated with enhanced cytokine production by dendritic cells, coupled with enhanced cross-presentation capacity (33). This interaction has beneficial effects across the immune system as a whole and is, therefore, regarded as instrumental in an inflammatory response. Furthermore, the downregulation of CD25 and CTLA-4 on T cells also indicates the immunological switch from suppressive to activation, in response to TG6002/5-FC therapy.

Functional T-cell responses to treatment were evident against TG6002 but, more importantly, against TAAs, suggesting an antigen-targeted cytotoxic effect against tumors. TCRβ sequencing revealed that a greater number of T-cell clones targeting both cancer antigens in general and specifically neoantigens, emerged within patients receiving higher doses of virus. Anti-cancer clonal T-cell expansion is associated with improved anti-cancer activity (34), where increased CD8 T-cell tumor infiltration was also observed in the highest-dose cohort upon treatment. As such, whether a T cell is activated toward a TAA/neoantigen or the virus itself may ultimately result in a parallel anti-tumor response against virus-infected cells within the tumor microenvironment.

These data, in summary, represent the first-in-human dose-escalation study using IHA delivery of a vaccinia virus. This treatment strategy directly targets tumor tissue and is associated with effective viral replication, expression and activity of the *FCU1* transgene, immune activation, and evidence for antitumor immune activity. Further assessment of IHA delivery of OV in immunotherapy-sensitive cancers is merited.

## Supplementary Material

Supplementary Figure S1 Immunophenotyping patient PBMCs

Supplementary Table S1 Patient demographics and disease summary

Supplementary Table S2 Representativeness of study participants

Supplementary Table S3 Treatment cohorts with patient trial/manuscript ID designation
