# Supplementary material for: A Phase I Clinical Trial of Intrahepatic Artery Delivery of TG6002 in Combination with Oral 5-Fluorocytosine in Patients with Liver-Dominant Metastatic Colorectal Cancer
Source: Clin Cancer Res. 2025 Jan 9;31(7):1243–56. doi: 10.1158/1078-0432.CCR-24-2498 (PMC11959272; doi:10.1158/1078-0432.CCR-24-2498)
Supplement: Supplementary Figure S1 — Immunophenotyping patient PBMCs [file ccr-24-2498_supplementary_figure_s1_suppfs1.pdf]

Supplementary Figure S1

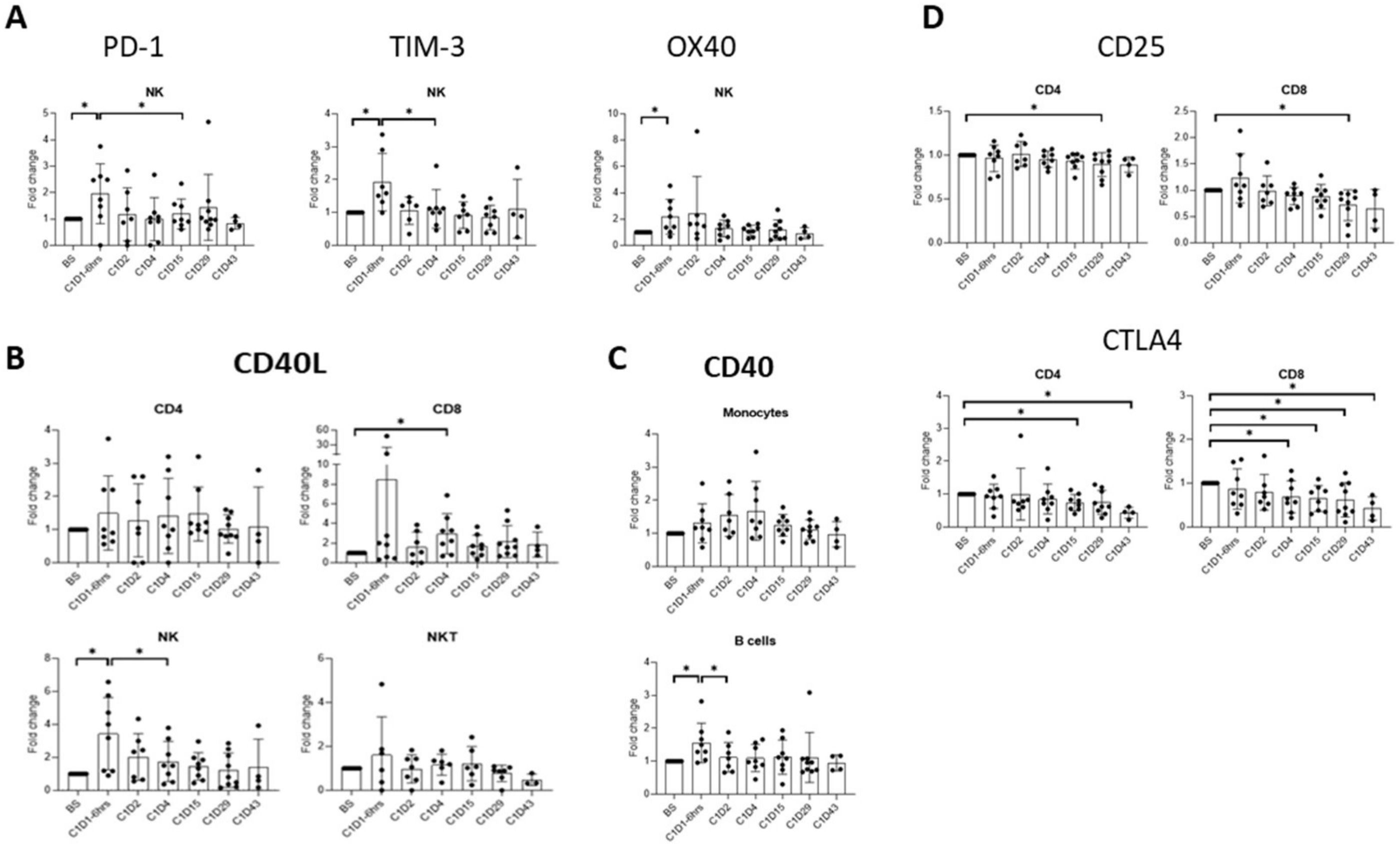

Supplementary Figure S1: Immunophenotyping of patient PBMCs.

Immunophenotyping of patient PBMCs indicates changes in expression of (A) PD-1, TIM-3 and OX-40 (B) CD40 and (D) CD25 and CTLA-4.

Relevant cell populations are depicted for each plot. Data is expressed as the mean fold change ± SEM; N=9 patients; *P<0.05, paired T test.
