# Supplementary material for: A Phase I Clinical Trial of Intrahepatic Artery Delivery of TG6002 in Combination with Oral 5-Fluorocytosine in Patients with Liver-Dominant Metastatic Colorectal Cancer
Source: Clin Cancer Res. 2025 Jan 9;31(7):1243–56. doi: 10.1158/1078-0432.CCR-24-2498 (PMC11959272; doi:10.1158/1078-0432.CCR-24-2498)
Supplement: Supplementary Table S1 — Patient demographics and disease summary [file ccr-24-2498_supplementary_table_s1_suppts1.pdf]

<table>
<tr><th colspan="6">Patient and Disease Demographics (n / %)</th></tr>
<tr><th colspan="2">Gender</th><th colspan="2">Age at ICF Signature</th><th colspan="2">ECOG Performance Status</th></tr>
<tr><td>Female</td><td>4 (26.7 %)</td><td>Mean</td><td>61 years</td><td>0</td><td>9 (60 %)</td></tr>
<tr><td>Male</td><td>11 (73.3 %)</td><td>Min-max</td><td>37 - 78 years</td><td>1</td><td>6 (40 %)</td></tr>
<tr><th colspan="4">Primary Location of Disease</th><th colspan="2">Time Since Initial Diagnosis</th></tr>
<tr><td colspan="2">Colon</td><td colspan="2">11 (73.3 %)</td><td>Mean (SD)</td><td>36.5 (23.8) months</td></tr>
<tr><td colspan="2">Rectum</td><td colspan="2">4 (26.7 %)</td><td>Min - max</td><td>8.1 – 89.9 months</td></tr>
<tr><th colspan="4">Stage at Initial Diagnosis</th><th colspan="2">Number of Antineoplastic Therapy Lines</th></tr>
<tr><td colspan="2">IIIB</td><td colspan="2">1 (6.7 %)</td><td>Mean (SD)</td><td>3.3 (1.72)</td></tr>
<tr><td colspan="2">IIIC</td><td colspan="2">2 (13.3 %)</td><td>Min - max</td><td>1.0 – 7.0</td></tr>
<tr><td colspan="2">IV</td><td colspan="2">12 (80 %)</td><td></td><td></td></tr>
<tr><th colspan="4">Total Number of Patients Screened</th><th colspan="2">Number of Screen Failures</th></tr>
<tr><td colspan="4">20</td><td colspan="2">5</td></tr>
<tr><th colspan="4">Reason for Screen Failures</th><th colspan="2">Patients Receiving at Least One Infusion</th></tr>
<tr><td colspan="2">Exclusion criteria: 2</td><td colspan="2">Inclusion criteria: 3</td><td colspan="2">15</td></tr>
<tr><th colspan="4">Patients Completing the Trial (13)</th><th colspan="2">Patients Not Completing Trial (2)</th></tr>
<tr><td colspan="2">Patients with disease progression</td><td colspan="2">12</td><td>Withdrawal due to adverse event</td><td>1</td></tr>
<tr><td colspan="2">Patients deceased</td><td colspan="2">1</td><td>Withdrawal due to palliative treatment</td><td>1</td></tr>
</table>

<u>Supplementary Table S1:</u> Patient demographics and disease summary.

ICF: informed consent form; ECOG PS: Eastern Co-operative Oncology Group Performance Status
