# Supplementary material for: A Phase I Clinical Trial of Intrahepatic Artery Delivery of TG6002 in Combination with Oral 5-Fluorocytosine in Patients with Liver-Dominant Metastatic Colorectal Cancer
Source: Clin Cancer Res. 2025 Jan 9;31(7):1243–56. doi: 10.1158/1078-0432.CCR-24-2498 (PMC11959272; doi:10.1158/1078-0432.CCR-24-2498)
Supplement: Supplementary Table S2 — Representativeness of study participants [file ccr-24-2498_supplementary_table_s2_suppts2.pdf]

Cancer type:	Colorectal cancer (CRC)
Considerations related to:	
Age	The median age at diagnosis for CRC cancer is 67. Older individuals are predominantly affected, with the majority of cases arising in people age 50 and above.
Sex	The lifetime risk of developing CRC is approximately 1 in 25 for females and 1 in 23 for males.
Race/ethnicity	In the US, CRC incidence is higher for Black Americans (41.9 per 100,000) compared to that of White Americans (37.0 per 100,000), with a Black:White incidence ratio of 1.13:1.
Geography	CRC is the third most common cancer worldwide. This accounts for approximately 10 % of all cases of cancer, making it the second leading cause of cancer-related deaths worldwide.
Overall representativeness of this study	The age distribution of patients within our study is slightly younger than the average age distribution of CRC in the literature, mean age of 61; range 37-78 years. The number of male and female participants in our study is 11 and 4, respectively, which equates to a greater number of males to the overall gender distribution. The race distribution is not available for our patients as the majority were not reported/recorded.

<u>Supplementary Table S2:</u> Representativeness of study participants.
