# Supplementary material for: A Phase I Clinical Trial of Intrahepatic Artery Delivery of TG6002 in Combination with Oral 5-Fluorocytosine in Patients with Liver-Dominant Metastatic Colorectal Cancer
Source: Clin Cancer Res. 2025 Jan 9;31(7):1243–56. doi: 10.1158/1078-0432.CCR-24-2498 (PMC11959272; doi:10.1158/1078-0432.CCR-24-2498)
Supplement: Supplementary Table S3 — Treatment cohorts with patient trial/manuscript ID designation [file ccr-24-2498_supplementary_table_s3_suppts3.pdf]

Cohort (dose)	Patient ID (trial)	Patient ID (manuscript)
1 ($1x10^6$ pfu)	0601-001	01
	0601-003	02
	0601-004	03
2 ($1x10^7$ pfu)	0601-005	04
	0102-001	05
	0601-007	06
3 ($1x10^8$ pfu)	0601-008	07
	0101-002	08
	0101-003	09
4 ($1x10^9$ pfu)	0101-004	10
	0101-005	11
	0101-007	12
	0101-006	13
	0101-008	14
	0601-011	15

Supplementary Table S3: Treatment cohorts with patient trial / manuscript ID designation.

Patient trial IDs and their associated manuscript IDs are shown, alongside relevant cohort and TG6002 dose
